# Antibacterial and antifungal activities and phytochemical profile of leaf extract from different extractants of *Ricinus communis* against selected pathogens

**DOI:** 10.1186/s13104-017-3001-2

**Published:** 2017-12-01

**Authors:** Jennifer Suurbaar, Richard Mosobil, Addai-Mensah Donkor

**Affiliations:** Department of Chemistry and Biochemistry, Faculty of Applied Sciences, University for Development Studies, Navrongo Campus, Tamale, Ghana

**Keywords:** Antibacterial, Antifungal, *Ricinus communis*, *Escherichia coli*, *Staphylococcus aureus*, *Pseudomonas aeruginosa*, *Klebseilla pneumoniae*, *Candida albicans*

## Abstract

**Objectives:**

*Ricinus communis* leaves are used in herbal preparations for treating candidiasis, skin and wound infections in Ghana. This study aimed at comparing the phytochemical profile of aqueous, methanol, petroleum ether, ethyl acetate and ethanolic extracts of the leaves of *Ricinus communis* and determine the growth inhibitory activities, bactericidal, bacteriostatic and fungicidal effects of the respective extracts on *Escherichia coli*, *Staphylococcus aureus*, *Pseudomonas aeruginosa*, *Klebsiella pneumonaie* and *Candida albicans*.

**Results:**

The aqueous, methanol and ethanol extracts were shown to contain most of the phytochemicals analyzed. All solvents extracts exhibited inhibitory activity against the growth of all microorganisms under study. The methanol extract showed highest zones of inhibition and was found to be statistically significant (*P* < 0.05) compared to other solvents extracts. All solvents extracts exhibited both bacteriostatic and bactericidal effects on the test organisms at varying concentration, with MIC values ranging from 3.13 to 25.0 mg/ml and MBCs were from 200 to 400 mg/ml. MFCs of *Candida albicans* was between 200 and 400 mg/l. Our data confirm the anti-bacterial and anti-fungal properties of *R. communis* and showed that the biologically relevant phytochemicals from the leaves of this plant can be extracted with the solvents aqueous, methanol and ethanol.

**Electronic supplementary material:**

The online version of this article (10.1186/s13104-017-3001-2) contains supplementary material, which is available to authorized users.

## Introduction

Plant kingdoms are the rich source of organic compounds, many of which have been used for medicinal purposes. There are many natural crude drugs from plants that have the potential to treat many disease and disorders and one of them is *Ricinus communis* [1, 2]. *Ricinus communis* is a species of flowering plant in the family, Euphorbiaceae. The parts of the plants used for medicinal purposes are the leaves, root, stem, fruits, complete aerial parts, the whole plant and flowers [3]. The plant is reported to contain antioxidant properties in its methanolic leaf extract [4, 5] anti-inflammatory activity [6], anti-diabetic activity [7] and antibacterial activity [4]. The plant has hepatoprotective effect [8] and has been used in the treatment of skin cancer [9].

A phytopharmacological review by Jena and Gupta in 2012, revealed that, *Ricinus communis* has proven to possess antimicrobial activities as they were used against dermatophytic and pathogenic bacterial strains *S. aureus*, *P. aeruginosa* as well as *K. pneumoniae* and *E. coli* [10]. Also, anti-fungal activity of the leaf was potent against *Candida albicans* [11]. The *Ricinus communis* possess wound healing activity due to the active constituent of castor oil which produce antioxidant activity and inhibit lipid peroxidation [12]. The leaves of *R. communis* are believed to be used in the form of a poultice or fomentation on sores, boils and swellings [3]. In this study, we validated the antimicrobial action of the extracts of the plant *R. communis* from Ghana using aqueous, methanol, petroleum ether, ethyl acetate and ethanolic solvents and also determined the bactericidal, fungicidal and bacteriostatic properties of the respective solvent extracts.

## Main text

### Materials and methods

#### Plant material

Plant material, *Ricinus communis* leaves were collected from different areas in Navrongo, Upper East Region, Ghana. The plant was identified and authenticated by a plant taxonomist at the herbarium of Ghana Herbaria, Northern Savanna Biodiversity; Savanna Herbarim. The voucher specimen was deposited with a number SH 720 in the herbarium.

#### Preparation of plant crude extracts

Plant material, *Ricinus commmunis* leaves were washed with distilled water and air dried at room temperature for 2 weeks. The leaves were ground into uniform powder.

The ethanol, methanol, petroleum ether, ethyl acetate and aqueous extracts were prepared by socking 100 g of the powdered plant materials in 1 l of each extractant at room temperature for 48 h. The extracts were filtered separately through whatman filter paper No 42 and concentrated using rotary evaporator (Heidoph 4001 efficient), warmed on water bath at 70 °C for the aqueous extract and temperature of 50 °C for ethanol, petroleum ether, ethyl acetate and methanol extracts, to obtain semi solid products.

#### Phytochemical screening

Phytochemical screening for the plant extracts were performed to determine the presence of tannins, saponins, terpenoids, polyuronoids, reducing sugars, flavonoids, alkaloids and anthraquinones using the method described elsewhere [12, 13].

#### Preparation of extracts concentrations from various extractants

The concentrations of the crude extracts obtained from the respective solvents were prepared using dimethylsulfuroxide (DMSO) to obtain concentrations of 200, 100, 50, 25, 12.5, 6.25, 3.125 and 1.5625 mg/ml for each extract.

#### Test organism

Disease-causing microorganism were taking into consideration and four bacteria and one fungus were considered. Selected bacterial species were *E. coli*, *S. aureus*, *P. aeruginosa* and *K. pneumoniae and C. albican as the fungus.* Clinical isolates of these microorganisms were obtained from the Microbiology Department of the Tamale Teaching Hospital in the Northern Region of Ghana, in the month of March 2016. Bacterial isolates were maintained at 2 and 8 °C on nutrient broth whilst the fungal isolates were maintained at 4 °C on potato dextrose agar.

#### Agar well diffusion assay

The modified agar well diffusion method described elsewhere [13] was employed.

#### Test for antifungal activity

In order to investigate the antifungal activity of the extracts, a micro dilution technique was used. The fungal spores were washed from the surface of agar plates with sterile 0.85% saline containing 0.1% Tween 80 (v/v). The spore suspension was adjusted with sterile saline to a concentration of approximately 1.0 × 10^7^ cfu/ml. The inocula were stored at 4 °C for further use. Dilutions of the inocula were cultured on solid potato dextrose agar to verify the absence of contamination and to check the validity of the inoculum.

#### Inoculum preparation for minimum inhibitory concentration (MIC) and minimum bactericidal concentrations (MBC)

Inocula were obtained from an overnight agar culture of the test organism. Inoculum for the MIC and MBC test was prepared by taking at least three to five well isolated colonies of the same morphology from agar plate culture. The top of each colony was touched with a sterile loop and the loop was transferred into a tube containing 5 ml of normal saline and then vortexed. The broth culture was incubated at 37 °C and monitored for approximately 4 h until it achieved the turbidity of 0.5 McFarland standard (1.5 × 10^8^ cfu/ml).

#### Determination of MBC and MIC

The tube diffusion method described elsewhere [13, 14] was employed for the determination of MBCs and MICs.

#### Determination of minimum fungicidal concentration (MFC)

Applying the method of [14], the minimum fungicidal concentrations (MFCs) were determined by subculturing of 2 μl from each of the wells showing no growth into microtiter plates containing 100 μl of broth per well and further incubation for 72 h at 28 °C. The lowest concentration with no visible growth was defined as MFC indicating 99.5% killing of the original inoculum. Commercial standards, Flucanazole (Sigma), was used as positive control (1–3000 μg/ml) and negative control (DMSO—99.9%) for fungus. All experiments were performed in duplicate and repeated three times for reproducibility.

### Statistical analysis

Means and standard error of the mean were calculated for the zones of inhibition measured for the two sets of experiments in each case. These means were statistically compared using the one-way ANOVA to determine if they were significantly different at *P* < *0.05.*

### Results and discussion

*Pseudomonas aeruginosa*, *Klebsiella pneumoniae*, *Escherichia coli* and many other β-lactamase producers have become a major clinical problem. Increased consideration has been focused on the usage of natural antimicrobial agents, especially from plant origins, due to their safety and efficacy as well as the fact that the majority of these plants are classified as generally recognized as safe. Indeed, natural products are used intensively as food preservatives, nutraceuticals as well as potential drugs for the treatment and prevention of various diseases and conditions including: cancer, cardiovascular disorders, aging and many others. Recently, for these reasons, global education have been conducted for the characterization, utilization and extraction of biological and pharmacological active compounds from plant origins.

In this study, the leaves of *Ricinus communis* were used, this is because, the leaves of the plant are mostly used in the treatment of wound infections, candidiasis and other skin diseases locally. For 100 g each in the different solvent the percentage yields were determined and were found to be 5.2, 6.0, 6.8, 7.2 and 8.3% for ethanol, aqueous, petroleum ether, ethyl acetate and methanol respectively. The phytochemical analysis of the different leaf extracts from the various extractants, aqueous, methanol and ethanol showed the presence of tanins, saponnins, terpenoids and flavonoids. Petroleum ether and ethyl acetate were devoid of tanins and flavonoids, probably resulting in low inhibitory activity against the pathogens. Most of these phytochemicals are the basis for plants medicinal properties and these are starting materials for production of new drugs today.

The extracts were found to be effective against the pathogens used in this study, which highlight the potential of herbal drugs and their possible use as local medicine. Utilising a concentration of 50 mg/ml of the crude extracts from the various extractants, methanol extract showed appreciably equal but higher inhibitory activity against all the bacteria used in this research compared to the other solvent extracts. The observed zones of inhibition for the methanol extract were 20 ± 2.82, 20 ± 0.71, 21 ± 2.12 and 24 ± 1.41 (mm) for *E. coli*, *S. aureus*, *P. aeruginosa* and *K. pneumoniae* respectively (Additional file 1). The ethyl acetate extract exhibited relatively lower inhibitory activity against the bacterial strains, with zones of 11.5 ± 0.71, 12.0 ± 1.41, 14.5 ± 2.12 and 14.5 ± 0.71 (mm) for *E. coli*, *S. aureus*, *P. aeruginosa* and *K. pneumoniae* respectively.

As the concentration of the crude extracts were raised to 100 mg/ml the methanol extract inhibitory activity against *E. coli* and *S. aureus* were approximately the same as that of the 50 mg/ml but inhibitory activity increased appreciably against *P. aeruginosa* and *K. pneumoniae.* The ethanol extract showed a significant higher inhibitory activity against *P. aeruginosa* with a zone of 24 ± 1.8 mm (Additional file 2). Similar trend was observed by raising the extract concentrations to 200 mg/ml. The antimicrobial activities of methanol, aqueous and ethanol extracts were comparable with that of amoxicillin, the standard antibiotic (Additional file 3) whilst the negative control (DMSO—99.99%) showed no inhibitory activity.

Phytochemicals such as, tannins, saponins, terpenoids, polyuronoids, reducing sugars, flavonoids, alkaloids and anthraquinones were all detected in methanol extract (Table 1). The high antibacterial activity in the methanolic extract may be due to the presence of high amount of tannins, flavonoids, and terpenoids. Tannins and flavonoids possesses similar mechanism by providing a source of stable free radical and also forms complex with nucleophilic amino acids in protein leading to the inactivation of the protein and loss of function, their potential antimicrobial effect is great as they probably target microbial cell of surface-exposed adhesins, cell wall polypeptides and membrane bound enzymes [15]. Terpenoids are for dissolution of the cell wall of microorganisms by weakening the membranous tissue [16]. Saponins have the ability to cause leakage of proteins and certain enzymes from the cell [17].

**Table 1:** Phytochemical profile of plant extracts

Solvents	Tannins	Saponins	Polyuronoids	Reducing sugars	Terpenoids	Flavonoids	Alkaloids	Anthraquinones
Aqueous	**+**	**+**	**+**	**+**	**+**	**+**	**−**	**−**
Ethanol	**+**	**+**	**+**	**−**	**+**	**+**	**−**	**+**
methanol	**+**	**+**	**+**	**+**	**+**	**+**	**+**	**+**
Petroleum ether	**−**	**−**	**−**	**−**	**+**	**−**	**+**	**−**
Ethyl acetate	**−**	**+**	**−**	**−**	**+**	**−**	**+**	**+**

+, presence; **−**, not detected

Both petroleum ether and ethyl acetate extracts were devoid of the phytochemicals mentioned above, justifying their lower inhibitory activity against the tested strains employed in the current research.

The minimum inhibitory concentration (MIC) and minimum bactericidal concentration (MBC) of *Ricinus communis* leaf extract on the isolated pathogens ranged from 3.13 to 25.0 mg/ml and MBCs were from 200 to 400 mg/ml (Table 2). The MICs depending on the microbe and the extract, greater sensitivity was observed in methanol, ethanol and aqueous extracts and the least sensitive was petroleum ether and ethyl acetate extracts where most of their MBCs were undetected. Jayaseelan and Jashothan reported in their study that methanol and ethanol extracts revealed lowest MIC value (5 mg/ml) against *S. aureus* and *E. coli* [18]. Another research group reported that methanol extract of *Ricinus communis* was found to be more active against *S. aureus*, *P. aeruginosa* and *K. pneumoniae* [19]. In this study the more susceptible test organisms to the methanol extract were *P. aeruginosa* and *K. pneumoniae*. A similar study conducted by Kensa and Yasmin showed that the more susceptible organism was *E. coli* [20], however, Chwukuka et al. showed that *Ricinus communis* leaf extract did not inhibit *E. coli* [21]. The differences observed may be due to the different extraction process and the difference in the susceptibilities of the clinical strains used.

**Table 2:** MIC and MBC and MFC (mg/ml) of *R. communis* extracts on the bacterial and fungal clinical isolates

Test organisms	Different solvents									
	Aqueous		Ethanol		Methanol		PET ether		Ethyl ace	
	MIC	MBC	MIC	MBC	MIC	MBC	MIC	MBC	MIC	MBC
*E. coli*	6.25	UD	6.25	UD	12.5	400	12.5	UD	25	400
*S. aureus*	3.13	300	25.0	400	6.25	300	12.5	300	12.5	200
*P. aeruginosa*	3.13	200	6.25	200	3.13	300	25	UD	25	UD
*K. pneumoniae*	12.50	400	6.25	200	6.25	400	25	UD	25	UD
*Fungus*		MFC		MFC		MFC		MFC		MFC
*C. albicans*	12.5	300	25	300	12.5	200	25	300	25	400

UD, undetected; PET, petroleum ether; Ethyl ace, ethyl acetate

**Fig. 1:**
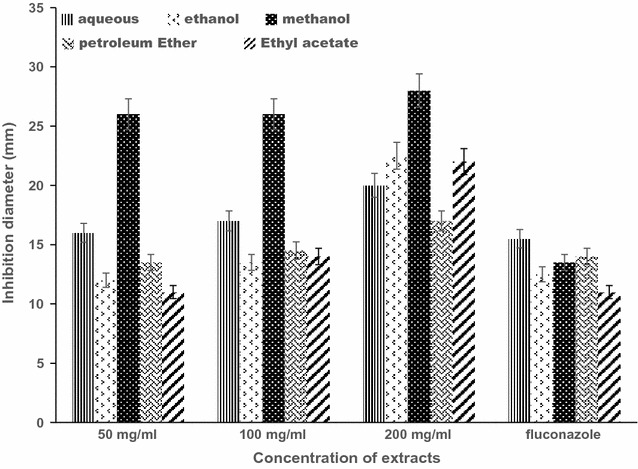
Representative antifungal activity of 50, 100 and 200 mg/ml of ethanol, methanol, aqueous, petroleum ether and ethyl acetate crude extracts of *Ricinus communis* against *C. albcans*. The data shown represent the average of three wells treated on the same day. The experiment was repeated twice and day-to-day variation was found to be within onefold of the presented data

Interestingly, the fungus, *C. albican* employed in this research was susceptible to all the extracts used, of which the methanol extract presented the highest inhibitory activity, followed by ethanol extract. Compared with the positive control (Fluconazole), the extracts exhibited significantly high inhibitory activity against the fungus with MFCs ranging from 200 to 400 mg/ml (Fig. 1) whilst the negative control (DMSO—99.99%) exhibited no inhibitory activity against the fungus. The results of this current research are in agreement with other findings supporting that most compounds in medicinal plants are more extracted in methanol [22].

### Conclusion

The extractants used have a major impact on inhibitory activity of the bioactive agents. In this study, methanol extract showed maximum antimicrobial activity, followed by ethanol and aqueous extracts. Petroleum ether and ethyl acetate showed the least antibacterial activity, suggestive of the active compounds having antimicrobial potential be extracted using appropriate solvent. This research gives a scientific validation to the fact that bioactive components in the plant *Ricinus communis* are extracted substantially in methanol and exhibited highly promising antibacterial and antifungal inhibitory activity.

### Limitation

Acquisition of reagents and chemicals was difficult and that caused hindrance in conducting other test of interest.

## Additional files

**Additional file 1.** Representative antibacterial activity of 50 mg/ml ethanol, methanol, aqueous, petroleum ether and ethyl acetate crude extracts of *Ricinus communis* against *E. coli*, *S. aureus*, *P. aeruginosa* and *K. pneumoniae*. The data shown represent the average of three wells treated on the same day. The experiment was repeated twice and day-to-day variation was found to be within onefold of the presented data.

**Additional file 2.** Representative antibacterial activity of 100 mg/ml ethanol, methanol, aqueous, petroleum ether and ethyl acetate crude extracts of *Ricinus communis* against *E. coli*, *S. aureus*, *P. aeruginosa* and *K pneumoniae*. The data shown represent the average of three wells treated on the same day. The experiment was repeated twice and day-to-day variation was found to be within onefold of the presented data.

**Additional file 3.** Representative antibacterial activity of 200 mg/ml ethanol, methanol, aqueous, petroleum ether and ethyl acetate crude extracts of *Ricinus communis* against *E. coli*, *S. aureus*, *P. aeruginosa* and *K pneumoniae*. The data shown represent the average of three wells treated on the same day. The experiment was repeated twice and day-to-day variation was found to be within onefold of the presented data.

## Abbreviations

*R. communis*: *Ricinus communis*
*K. pneumoniae*: *Klebsiella pneumoniae*
*E. coli*: *Escherichia coli*
*P. aeruginosa*: *Pseudomonas aeruginosa*
*S. aureus*: *Staphylococcus aureus*
*C. albicans*: *Candida albicans*
MIC: minimum inhibitory concentration
MBC: minimum bactericidal concentration
MFC: minimum fungicidal concentration
STD: standard deviation
